# The patient’s sex determines the hemodynamic profile in patients with Cushing disease

**DOI:** 10.3389/fendo.2023.1270455

**Published:** 2023-10-11

**Authors:** Agnieszka Jurek, Paweł Krzesiński, Beata Uziębło-Życzkowska, Przemysław Witek, Grzegorz Zieliński, Anna Kazimierczak, Robert Wierzbowski, Małgorzata Banak, Grzegorz Gielerak

**Affiliations:** ^1^ Department of Cardiology and Internal Medicine, Military Institute of Medicine – National Research Institute, Warsaw, Poland; ^2^ Department of Internal Medicine, Endocrinology, and Diabetes, Medical University of Warsaw, Warsaw, Poland; ^3^ Department of Neurosurgery, Military Institute of Medicine – National Research Institute, Warsaw, Poland

**Keywords:** Cushing disease, impedance cardiography, hemodynamic profile, cardiovascular complications, echocardiography

## Abstract

**Background:**

Cushing disease (CD) may lead to accelerated cardiovascular remodeling and increased mortality. There are suspected differences in the mechanism of cardiovascular dysfunction between males and females with CD. The purpose of this study was to assess the effect of patient sex on the hemodynamic profile assessed *via* impedance cardiography and echocardiography in patients newly diagnosed with CD.

**Material and methods:**

The 54 patients newly diagnosed with CD (mean age 41 years; 77.8% of females) who were included in this prospective clinical study underwent impedance cardiography to assess specific parameters (including systemic vascular resistance index [SVRI], total arterial compliance index [TACI], Heather index [HI], stroke index [SI], cardiac index [CI], velocity index [VI], and acceleration index [ACI]) and transthoracic echocardiography to assess heart chamber diameters and left ventricular systolic and diastolic function.

**Results:**

Males with CD exhibited higher afterload, with higher SVRI (3,169.3 ± 731.8 vs. 2,339.3 ± 640.8 dyn*s*cm^-5^*m² in males and females, respectively; p=0.002), lower TACI (0.80 ± 0.30 vs. 1.09 ± 0.30 mL/mmHg*m^2^; p=0.008), and lower hemodynamic parameters of left ventricular function, with lower HI (9.46 ± 2.86 vs. 14.1 ± 5.06 Ohm/s^2^; p=0.0007), lower VI (35.1 ± 11.9 vs. 44.9 ± 13.1 1*1000^-1^*s^-1^; p=0.009), lower SI (36.5 ± 11.7 vs. 43.6 ± 9.57 mL/m^2^; p=0.04), lower CI (2.36 ± 0.46 vs. 3.17 ± 0.76 mL*m^-2^*min^-1^; p=0.0009), and lower ACI (50.4 ± 19.8 vs. 73.6 ± 25.0 1/100/s^2^; p=0.006). There were no significant differences between the sexes in left ventricular systolic or diastolic function assessed by echocardiography.

**Conclusion:**

In comparison with females with CD, males with CD have a less favorable hemodynamic profile, with higher afterload and worse left ventricular function. Sex differences in cardiovascular system function should be taken into consideration in designing personalized diagnostic and therapeutic management of patients with CD.

## Introduction

Cushing disease (CD) is a rare endocrine disorder associated with excess adrenocorticotropic hormone (ACTH) secretion by a pituitary adenoma. Continued exposure to hypercortisolemia leads to hemodynamic dysfunction associated with accelerated cardiovascular remodeling, which ultimately produces multiple complications and increases mortality (1). The key factor contributing to cardiovascular disease in patients with CD is exacerbated inflammation as a result of overweight, abdominal obesity, hypercortisolism, insulin resistance, high levels of resistin, oxidized low-density lipoproteins (oxLDLs), triglycerides, C-reactive protein, and high leukocyte counts (2).

The mechanisms of cardiovascular dysfunction may be different in males and females with CD. There have been several studies that showed differences in cardiovascular function between the sexes in patients with CD (3–8), with the affected males believed to be more susceptible to cardiovascular complications than females (3, 4). This difference can be only partly explained by the protective cardiovascular effect of estrogens in premenopausal women. The question whether medical treatment in patients with CD (particularly those with concomitant hypertension [HTN]) should be more personalized and sex-dependent remains without a definitive answer. Therefore, there is a need for noninvasive methods, which would allow detailed assessment of cardiovascular function in patients of the two sexes diagnosed with CD, in order to initiate personalized treatment and, ultimately, to reduce the cardiovascular risk. Based on earlier reports (9–14), we hypothesized that the use of impedance cardiography (ICG), which provides a noninvasive means of assessing hemodynamic parameters, such as arterial stiffness and cardiac function, will illustrate the potential differences between male and female patients with CD in terms of cardiovascular dysfunction mechanisms. Such findings would demonstrate a substantial benefit of this technique in addition to those offered by the commonly used echocardiography. Consequently, the purpose of this study was to compare the hemodynamic profiles of females and males newly diagnosed with CD.

## Materials and methods

### Study population

A total of 54 patients newly diagnosed with CD, without clinically significant concomitant conditions, were included in this observational prospective study. The mean age of the study participants was 41 years, 64.8% of whom were diagnosed with HTN; females constituted 77.8% of the study population.

CD had been diagnosed based on the most recent guidelines on the diagnosis and management of CD. The diagnostic criteria included symptoms of hypercortisolemia and standard hormonal criteria: elevated urinary free cortisol (UFC), elevated serum cortisol levels at 8.00 a.m., loss of cortisol circadian rhythm, increased or detectable plasma ACTH levels at 8.00 a.m., and no reduction serum cortisol levels to <1.8 mg/dL during an overnight dexamethasone suppression test (1 mg of dexamethasone administered at midnight). A pituitary etiology of CD was confirmed by decrease in serum cortisol levels or an over 50% reduction in urinary free cortisol (UFC) in a 2-day (2 mg every 6 hours) high-dose dexamethasone suppression test (HDDST) or positive corticotropin-realeasing hormone (CRH) stimulation test (100 μg i.v.). Magnetic resonance imaging of the pituitary gland was performed in all patients, which confirmed the presence of a pituitary tumour. If the results of the hormonal dynamic tests or the MRI results were inconclusive or in the case of microadenomas < 6 mm in size found on MRI, the diagnostic tests were extended to include sampling of the inferior petrosal sinus (15–17).

In addition, plasma levels of ACTH, follicle-stimulating hormone (FSH), luteinizing hormone (LH), and thyroid-stimulating hormone (TSH) were analyzed. The study patients were not taking drugs that affect the function of the pituitary-adrenal axis. Thus, neither steroidogenesis inhibitors nor estrogen/testosterone therapy affected the results of the haemodynamic profile assessed by impedance cardiography. None of our patients had been pregnant and had not given birth in the last five years. Seven patients had become pregnant and given birth in the past. In the study group, infertility was the main problem among patients of reproductive age. Tests to detect possible disorders of carbohydrate metabolism, such as impaired fasting glucose (IFG), impaired glucose tolerance (IGT), and type 2 diabetes mellitus (T2DM), were also conducted. The study patients, all of whom had CD, had not been on any medications that may have affected the hypothalamic–pituitary–adrenal axis and, consequently, the assessed hemodynamic parameters.

Our study was conducted in accordance with the principles of Good Clinical Practice (GCP) and the Declaration of Helsinki and was approved by the local ethics committee at the Military Institute of Medicine – National Research Institute in Warsaw, Poland (approval No. 76/WIM/2016). All patients provided their written informed consent to participate in this study. The study was funded by the Ministry of Science and Higher Education/Military Institute of Medicine – National Research Institute in Warsaw, Poland (grant No. 453/WIM).

### Exclusion criteria

The study exclusion criteria were: (1) coronary artery disease; (2) heart failure with mid-range ejection fraction (HFmrEF) or heart failure with reduced ejection fraction (HFrEF); (3) history of pulmonary embolism, (4) history of stroke or transient ischemic attack (TIA); (5) chronic obstructive pulmonary disease (COPD); (6) respiratory failure (decreased partial pressure of oxygen (PaO_2_) <60 mmHg and/or increased partial pressure of carbon dioxide (PaCO_2_) >45 mmHg); (7) history of head trauma; (8) pregnancy; (9) lack of consent.

### Clinical examination

The clinical examination was performed with a particular focus on any risk factors indicating cardiovascular dysfunction (including history of cardiovascular symptoms, concomitant conditions, cardiovascular disease in the family, smoking, or disorders of carbohydrate metabolism; and measurements of patient’s height, body mass index (BMI), blood pressure (BP), systolic BP (SBP), diastolic BP (DBP), and heart rate (HR). BP measurements were conducted based on the European Society of Cardiology (ESC) guidelines, with the use of an automatic blood pressure monitor (Omron M4 Plus, Japan) (18).

### Impedance cardiography

Impedance cardiography is one noninvasive and well-validated tool for assessing cardiovascular hemodynamics based on the phenomenon of impedance variation of a given body segment related to blood flow in large arterial vessels. Blood circulating in the vascular system is characterised by a relatively low impedance in relation to the surrounding tissues, and its variability over time allows the assessment of many important haemodynamic parameters (12, 19–23).

Hemodynamic parameters were measured *via* ICG with a *Niccomo*™ device (Medis, Ilmenau, Germany) during a 10-minute resting examination in a supine position. Analysis of the obtained ICG records (*Niccomo Software*) included the mean values of such hemodynamic parameters as: HR [bpm]; SBP [mmHg]; DBP [mmHg]; thoracic fluid content (TFC [1/kOhm] = 1000/Z0, where Z0 is baseline thoracic impedance); stroke volume (SV [mL]) based on the formula SV = VEPT ×(dZmax/Z0) × LVET, where VEPT is the volume of electrically participating tissue, calculated from the subject’s weight, height, and sex, dZmax is the maximum change in thoracic impedance, Z0 is baseline thoracic impedance, and LVET is the left ventricular ejection time; stroke index (SI [mL/m^2^]); cardiac output (CO [mL] = SV × HR); cardiac index (CI [mL*m^-2^*min^-1^]); acceleration index (ACI [1/100/s^2^], which describes peak blood flow acceleration in the aorta; velocity index (VI [1*1000^-1^*s^-1^]); Heather index (HI [Ohm/s^2^], based on the formula HI = dZmax× TRC, where TRC is the time interval between the electrocardiographic R-peak and the C-point on the ICG wave); systemic vascular resistance (SVR [dyn*s*cm^-5^]); SVR index (SVRI [dyn*s*cm^-5^*m²]); total arterial compliance index (TACI [mL/mmHg] = SV/pulse pressure [mL/mmHg*m^2^]).

### Echocardiography

Echocardiographic examinations were conducted according to the current American Heart Association standards (a Vivid E 95 device, GE Healthcare, USA) (24). The evaluated parameters included heart chamber diameters (left atrial [LA] diameter, left ventricular end-diastolic diameter [LVEDD], right-ventricular end-diastolic diameter [RVEDD], ascending aortic diameter [AoA]); wall thickness measures (interventricular septal end-diastolic diameter [IVSd], left ventricular mass index [LVMI], left ventricular hypertrophy [LVH]); left ventricular (LV) systolic function (including LV ejection fraction [LVEF] calculated with the Simpson method and global longitudinal strain [GLS], evaluated by speckle-tracking echocardiography; and LV diastolic function (including the early-to-late-diastolic mitral inflow velocity ratio [E/A] assessed with pulsed wave Doppler, as well as the early-diastolic mitral annular velocity [e’] and the peak early diastolic mitral inflow velocity-to-early diastolic mitral annular velocity ratio [E/e’], assessed with tissue Doppler).

### Statistical analysis

Statistical analysis was conducted with *Statistica 12.0* (*StatSoft Inc., Tulsa, USA*). Normality of data was assessed visually and with the Kolmogorov–Smirnov test. Continuous variables were expressed as means ± standard deviations (SDs), whereas discrete (qualitative) variables were expressed as absolute values (n) and percentages (%). The distribution of continuous variables was assessed visually and with the Shapiro–Wilk test. The differences in the absolute values of continuous variables between males and females were compared with a t-test for normally distributed data and the Mann–Whiney U test for non-normally distributed data. P-values of <0.05 were considered statistically significant.

## Results

### Demographic and clinical characteristics of the CD population

Detailed characteristics of patients with CD have been presented in **Table 1**. The study group comprised mostly females (77.8%). Thirty-five patients (64.8%) had been diagnosed with HTN, and all of them were receiving medical treatment, usually with one or two antihypertensive drugs. Mean blood pressure values were 126/83 mmHg. Nine patients had a previous diagnosis of dyslipidaemia and were treated with statins. The mean LDL cholesterol values in the statin-treated group were 55.5 mg/dl. Thus, we wanted to exclude a potential influence of HTN and dyslipidaemia on the results obtained. The mean BMI was nearly 30 mg/m^2^. Twenty (37%) out of the 54 patients with CD had T2DM; 15 of those were treated with metformin, five with metformin and insulin, and one with insulin alone. Forty-three of the patients with CD had normal anterior pituitary function. Eleven patients with an invasive corticotropic tumor had TSH deficiency; however, it was well controlled with a stable-dose L-thyroxin regimen.

**Table 1:** Basic characteristics of the study group.

Variables	mean ± SD/n (%)
age (years)	41.1 ± 13.5
females	42 (77.8)
BMI (kg/m^2^)	29.7 ± 6.5
HR (office) (bpm)	71.9 ± 11.4
SBP (office) (mm Hg)	126 ± 17
DBP (office) (mm Hg)	83 ± 12
LVEF (%)	66.7 ± 4.2
hemoglobin (g/L)	14.5 ± 1.3
creatinine (mg/dL)	0.87 ± 0.27
Comorbidities	
hypertension	35 (64.8)
dyslipidemia	9 (16.7)
diabetes mellitus	20 (37.0)
current smoking	6 (11.1)
Symptoms	
exercise intolerance	40 (74.1)
dyspnea on exertion	18 (33.3)
dyspnea at rest	8 (14.8)
dizziness	19 (35.2)
syncope	4 (7.4)
heart palpitations	23 (42.6)
peripheral edema	29 (53.7)

BMI, body mass index; DBP, diastolic blood pressure; HR, heart rate; LVEF, left ventricular ejection fraction; SBP, systolic blood pressure; SD, standard deviation.

### A comparison between males and females with CD in terms of basic clinical characteristics

A comparison of basic characteristics between males and females with CD has been presented in **Table S1**. These two subgroups showed no differences in terms of age, BMI, or the rates of hypertension, dyslipidemia, T2DM, or smoking. Moreover, the two sex subgroups showed no significant differences in terms of clinical manifestations.

### A comparison between males and females with CD in terms of two-dimensional echocardiographic parameters

The only differences between male and female patients identified with echocardiography were RVEDD and LA diameter, which were due to constitutional differences between the sexes – **Table 2**. There were no significant differences between the sexes in terms of LVEF, GLS, or parameters of LV diastolic dysfunction.

**Table 2:** Comparison between males and females – hemodynamics.

Variables	Males (n = 12) mean ± SD/n (%)	Females (n=42) mean ± SD/n (%)	p-value
Echocardiography			
RVEDD [mm]	31.9 ± 2.86	27.8 ± 3.71	0.001
LVEDD [mm]	48.7 ± 3.89	46.9 ± 4.36	0.168
LA diameter [mm]	38.7 ± 3.32	35.6 ± 3.93	0.024
AoA [mm]	32.1 ± 3.75	29.8 ± 3.57	0.059
LVMI [g/m^2^]	105.8 ± 22.2	92.1 ± 21.8	0.053
LVEF [%]	65.3 ± 3.57	65.9 ± 4.33	0.454
GLS [%]	18.3 ± 2.41	18.0 ± 2.34	0.552
E/A [-]	0.94 ± 0.27	1.13 ± 0.34	0.086
avg e’ [cm/s]	9.14 ± 2.67	9.66 ± 3.36	0.748
E/e’ [-]	7.00 ± 1.80	7.83 ± 2.08	0.234
Impedance cardiography			
HR [bpm]	67.6 ± 10.7	72.5 ± 12.2	0.230
SBP [mmHg]	132.7 ± 17.7	123.7 ± 16.5	0.102
DBP [mmHg]	84.5 ± 10.7	81.9 ± 11.9	0.670
MBP [mmHg]	95.6 ± 10.3	92.7 ± 12.5	0.466
PP [mmHg]	48.4 ± 13.6	41.8 ± 10.2	0.158
SI [mL/m^2^]	36.5 ± 11.7	43.6 ± 9.57	0.041
CI [mL*m^-2^*min^-1^]	2.36 ± 0.46	3.17 ± 0.76	0.0009
VI [1*1000^-1^*s^-1^]	35.1 ± 11.9	44.9 ± 13.1	0.009
ACI [1/100/s^2^]	50.4 ± 19.8	73.6 ± 25.0	0.006
HI [Ohm/s^2^]	9.46 ± 2.86	14.1 ± 5.06	0.0007
SVRI [dyn*s*cm^-5^*m²]	3,169.3 ± 731.8	2,339.3 ± 640.8	0.002
TACI [mL/mmHg*m^2^]	0.80 ± 0.30	1.09 ± 0.30	0.008
TFC [1/kOhm]	26.9 ± 2.74	25.2 ± 4.12	0.148

AoA, ascending aortic diameter; ACI, acceleration index; CI, cardiac index; DBP, diastolic blood pressure; e’, early diastolic mitral annular velocity, E/A, early-to-late-diastolic mitral inflow velocity ratio, E/e’, early mitral inflow-to-early mitral annular velocity ratio, GLS, global longitudinal strain; HI, Heather index; HR, heart rate; LA diameter, left atrial diameter; LVEDD, left ventricular end-diastolic diameter; LVEF, left ventricular ejection fraction; LVMI, left ventricular mass indexed to body surface area; MBP, mean blood pressure; PP, pulse pressure; RVEDD, right ventricular end-diastolic diameter; SBP, systolic blood pressure; SD, standard deviation; SI, stroke index; SVRI, systemic vascular resistance index; TACI, total arterial compliance index; TFC, thoracic fluid content; VI, velocity index.

### A comparison between males and females with CD in terms of the hemodynamic parameters assessed with ICG

Mean BP values in both subgroups were normal. Despite there being only slight differences in basic hemodynamic parameters (HR, SBP, and DBP), a comparative analysis of the remaining ICG parameters revealed more pronounced differences between the sexes – **Table 2**. ICG showed that male patients had stiffer arteries, as evidenced by a higher SVRI (p=0.002) and a lower TACI (p=0.008) as well as lower hemodynamic parameters of LV function, including a lower HI (p=0.0007), a lower VI (p=0.009), a lower SI (p=0.04), a lower CI (p=0.0009), and a lower ACI (p=0.006). No significant differences between the sexes were observed in terms of TFC.

## Discussion

The comprehensive assessment of hemodynamic parameters with the use of ICG revealed the hemodynamic profiles of male patients with CD to be different from those of females with CD. Our analysis of patients newly diagnosed with CD also showed significant differences between males and females in terms of hemodynamic dysfunction parameters, despite normal LV systolic and diastolic function and optimal BP control. The patients from both study subgroups were comparable in terms of their basic characteristics (which eliminated potential effects of patient age, BP, HR, or BMI) and symptoms, such as dyspnea, palpitations, or pain in the chest. Since the patients included in this study had neither clinically overt cardiovascular dysfunction nor any serious comorbidities, we were able to obtain a comprehensive hemodynamic profile in males and females with CD, which indicated sex differences in the pathophysiology of CD.

Neither basic nor clinical characteristics of our study population differed from those reported in other studies on cardiovascular disorders in patients of either sex with CD (3–8). The preponderance of females among patients with CD may be associated with estrogen receptor expression on ACTH-secreting pituitary adenomas (3, 4). Sex steroids have been suggested to be a contributing factor in the development of ACTH-secreting pituitary tumors (25). Analysis of molecular features characterizing ACTH-secreting pituitary adenomas collected from male and female patients with CD revealed differences between the sexes, indicating a need for their analysis in each sex separately (26). We would like to emphasize that the detailed analysis of hemodynamic parameters conducted in our study with the use of ICG with study group stratification by sex is one of the first such attempts and it may cast new light on CD pathophysiology.

ICG assessment of hemodynamic parameters demonstrated a significantly worse cardiac function, decreased myocardial contractility, increased vascular resistance, and decreased artery compliance in males with CD, in comparison with those in females with CD. These findings confirm an unfavorable hemodynamic profile in males with CD – characterized by pronounced vasoconstriction and impaired LV function. Subclinical hemodynamic changes indicate an additive effect of long-term hypercortisolemia on cardiovascular function, which is significantly greater in males than females with CD. The data obtained in our study are consistent with those reported by other authors, who demonstrated that, in comparison with females, males with CD have a higher cardiovascular risk (HTN, anemia, hypercoagulable states, hypokalemia, arrhythmias, dyslipidemia, high HbA1c levels, increased BMI), lower quality of life (low libido, sexual dysfunction, myopathy, stretch marks, lumbar osteoporosis, fractures, nephrolithiasis), worse biochemical parameters (higher serum ACTH, serum cortisol, and UFC levels), and worse postoperative prognosis (lower rates of postoperative normalization of cortisol levels and higher risk of recurrence) (3–8, 25, 26). Moreover, the hormone tests used to assess pituitary disease etiology showed a lower sensitivity in male patients with CD, which caused males to more often undergo invasive procedures, such as petrosal sinus sampling, in order to establish a definitive diagnosis. Moreover, pituitary adenomas in males with CD were more difficult to visualize with MRI than those in females, which posed additional diagnostic challenges and delayed treatment in male patients (1, 3). These facts may be what contributes to delayed CD diagnosis in male patients and to the predominance of females among patients diagnosed with CD (3, 5).

The fact that male patients with CD had higher serum ACTH, cortisol, and UFC levels at the time of diagnosis suggested a higher secretory activity of pituitary adenomas in males in comparison with females (3–5). Osteoporosis (leading to fractures) and anemia were also more common in male than female patients with CD, which may be partly due to patients themselves delaying the diagnostic process (7). Apart from increased BMI, males with CD tend to additionally have fatty liver and hepatic dysfunction (8). HTN was reported to contribute to the increased mortality associated with a higher cardiovascular risk in males with CD. The duration of hypercortisolemia seems to correlate with the development of HTN in patients with CD. Possibly, ACTH not only induces the release of aldosterone but also inhibits 11β-hydroxysteroid dehydrogenase (11β-HSD) which may potentiate the effect of cortisol on mineralocorticoid receptors. Hypokalemia and HTN are strongly associated with overall CD mortality; therefore, males with CD tend to have a poorer prognosis than females (8). Moreover, males with CD have a prolonged QT interval, which may be related to a higher risk of ventricular arrhythmias. This may be due both to low testosterone level and to hypokalemia (27). Moreover, males with CD had a less favorable postoperative course of the disease (4), with considerably higher postoperative (at six months after surgery) cortisol levels and a higher rate of disease recurrence, which suggests that this subgroup of patients requires a careful long-term follow-up, even if the surgery seems to have been successful (6).

Most authors emphasize the need to differentiate the diagnostic process and treatment in CD depending on patient sex, which is due to the much higher risk of cardiovascular complications in males associated with difficulties in hormonal and imaging diagnosis, which lead to delayed treatment initiation (3–5). Some, though not many, authors present a different opinion by pointing out that male patients with ACTH-dependent Cushing syndrome seem to have a different clinical presentation in terms of symptoms and biochemistry; however, this should not affect further diagnostic strategy, treatment, or surgical outcomes. Thus, patients require no different diagnostic or therapeutic strategies with respect to their sex (7).

Our observations revealed significant differences between the sexes in cardiovascular hemodynamic function in patients newly diagnosed with CD. These differences may affect the personalized treatment approach. Earlier recognition of cardiovascular complications and earlier introduction of preventive measures in patients with CD, depending on their sex, may lower the cardiovascular risk. Personalizing the treatment based on the hemodynamic profile and the patient’s sex may be associated not only with better BP control, but also with higher chances of achieving a normal hemodynamic profile. The differences between the sexes in hemodynamic phenotypes may help develop more personalized therapeutic approaches for patients with CD. Current guidelines stress the necessity of personalized therapy (18).

Sex-based differences in cardiovascular system function have been already described in patients with HTN (28–30). Females with HTN exhibited a different hemodynamic profile when assessed with ICG in comparison with that of males, namely lower large artery compliance, with better LV function as a pump. This may be explained by a considerable role of estrogens in neutralizing the unfavorable features of the female hemodynamic profile and protecting women against adverse cardiovascular events. Estrogens reduce adrenergic-mediated vasoconstriction, increase nitric oxide release, and affect the renin–angiotensin–aldosterone system by inhibiting rein production and AT1 receptor expression. Males do not have this protection. At the same time, estrogens increase the expression of AT2 receptors, which play an important role in BP regulation in women. Moreover, males with HTN are more vulnerable to endothelin 1, which is a vasoconstrictor (28, 31–35). Another study showed that young women have higher mean CI and lower SVRI values than men of the same age; however, these hemodynamic differences between the sexes disappear after menopause (29). Another study assessed the relationship between LV diastolic function and the hemodynamic profile assessed with the use of ICG. LV diastolic dysfunction and arterial stiffness have been reported in young and middle-aged patients with HTN. The fact that patient’s sex may affect cardiovascular hemodynamics should be taken into account in therapeutic strategies (30). Moreover, other studies demonstrated significant differences in CO between the sexes (36, 37). Female hearts were reported to react to pressure overload by an increase in mitochondrial gene expression, whereas male hearts responded by an increased expression of genes responsible for protein and extracellular matrix synthesis, which shows that genetic analyses also provide a molecular basis for sex differences in cardiovascular function (38). Increased arterial stiffness, which is characterized by an increased SVRI and decreased TAC, has been reported to increase the cardiovascular risk (36–38). These hemodynamic changes were observed in the male subgroup of our study. As a result, males may be at a higher risk of serious complications (i.e. myocardial infarction, stroke, or HF). As a result of long-term exposure to increased, prolonged pressure overload and long-term effects of chronic hypercortisolemia, males are more predisposed to develop HTN, HF, and coronary artery disease, even if there are no detectable imaging study abnormalities at the time of diagnosis. Already at the time of CD diagnosis, the heart of a male with CD has been working against stiff vessels in order to maintain an adequate LVEF. In response to lower arterial compliance, LV undergoes remodeling, and this may lead to reduced myocardial perfusion and decreased exercise tolerance.

The echocardiographic differences between the sexes in absolute heart chamber diameters and LV mass are a result of the constitutional differences. The observed tendency (p = 0.053) for higher prevalence of LVH in males suggests the existence of certain distinct features characterizing the male subpopulation, since the rates of LVH in the general population are equally distributed between the sexes. The echocardiographic assessments in our study group showed no significant differences in LV function. Despite normal LV systolic and diastolic function illustrated by echocardiography, ICG results indicated subclinical hemodynamic differences. Our study showed that the parameters of cardiac function as a pump, namely SI, VI, CI, and ACI, were lower in males with CD than in females with CD. This may be due to accelerated, subclinical, hypercortisolemia-induced myocardial remodeling associated with concentric LVH (39, 40). The observed changes are associated not only with myocardial hypertrophy due to pressure overload, since cortisol also directly leads to myocardial fibrosis (40). Myocardial remodeling and dysfunction may increase considerably in patients with CD with concomitant HTN and hypercortisolemia. The abnormal hemodynamic profile in males with CD observed in our study may lead to hemodynamic LV dysfunction and development of symptomatic HF (39, 41, 42). Females have been reported to have better echocardiographic LV parameters (including higher LVEF) than males (43, 44). Abnormal GLS in echocardiography was associated with a hemodynamic profile similar to that observed in the subgroup of males with CD (low CI, high SVRI) (45). Early LV dysfunction in the form of abnormal GLS and LV diastolic dysfunction were observed already at early stages of pituitary disease (46).

These study results have important practical applications. Routine BP measurements and routine imaging studies may be insufficient to identify sex-dependent hemodynamic abnormalities. Cardiovascular hemodynamic differences between the sexes may be responsible for various responses to medical treatment. This indicates that ICG has an added value in assessing cardiovascular hemodynamic parameters in patients with CD.

### Clinical implications

Our observations revealed sex differences in the cardiovascular hemodynamic parameters of patients with CD, which may affect antihypertensive treatment personalization and hinder attempts to introduce early prevention, particularly in males with CD. In light of the fact that antihypertensive treatment personalization based on an individual hemodynamic profile may lead to better BP control, such differences between the sexes in hemodynamic phenotypes may help identify more personalized therapeutic approaches. Current guidelines emphasize the need for personalized treatment, which suggests the direction of future studies. Moreover, early introduction of preventive treatment in males with CD based on their hemodynamic profiles may help prevent the development of cardiovascular complications, including HTN, HF, or coronary artery disease, even before they become symptomatic, which may be clinically significant and may contribute to reducing mortality in this patient subgroup. Studies show that, due to the particularly unfavorable hemodynamic profile in males with CD, first-line preventive and antihypertensive measures should include vasodilators and cardioprotective agents, such as angiotensin converting enzyme (ACE) inhibitors, angiotensin receptor blockers (ARB), and calcium channel blockers (CCB) (46, 47). Low TFC with normal LVEF in men with CD indicates that diuretics should not be the treatment of choice in antihypertensive therapy.

### Study limitations

One limitation of our study was a relatively small sample size. This was dictated by a low prevalence of CD in the general population and a prospective nature of the study. The exclusion criteria further limited the study group only to patients newly diagnosed with CD without serious comorbidities or clinically significant cardiovascular dysfunction that might affect study results. Many patients with CD are already affected by multiple comorbidities at the time of their diagnosis, which makes patients newly diagnosed with CD but with no serious concomitant conditions even scarcer. Importantly, only young or middle-aged males and females with CD without any serious comorbidities or overt cardiovascular dysfunction were included in our study. This special, highly selected group of patients exhibited distinctive, subclinical hemodynamic changes already at the early stage of CD. If left untreated, these hemodynamic changes may lead to serious cardiovascular complications. The potential effect of concomitant HTN (even despite good BP control) and the use of antihypertensive treatment should be also considered in study result interpretation. In addition, the difference between cortisol-induced cardiovascular changes in different sexes should be clearly distinguished from physiological sex differences in cardiovascular disease, especially in premenopausal women.

## Conclusions

Males with CD, in comparison with their female counterparts, are characterized by a less favorable hemodynamic profile, with increased afterload and decreased LV function. Sex differences in cardiovascular function should be considered in personalizing the diagnostic and therapeutic approach to patients with CD.

## Data availability statement

The original contributions presented in the study are included in the article/**Supplementary Material**. Further inquiries can be directed to the corresponding author.

## Ethics statement

The studies involving humans were approved by Ethics Committee at Military Institute of Medicine National Research Institute. The studies were conducted in accordance with the local legislation and institutional requirements. The participants provided their written informed consent to participate in this study.

## Author contributions

AJ: Conceptualization, Data curation, Formal Analysis, Funding acquisition, Investigation, Methodology, Project administration, Resources, Software, Supervision, Validation, Visualization, Writing – original draft, Writing – review & editing. PK: Conceptualization, Funding acquisition, Investigation, Methodology, Project administration, Supervision, Writing – original draft, Writing – review & editing. BU: Conceptualization, Data curation, Investigation, Resources, Supervision, Writing – review & editing. PW: Conceptualization, Data curation, Investigation, Methodology, Resources, Supervision, Writing – review & editing. GZ: Conceptualization, Data curation, Investigation, Supervision, Writing – review & editing. AK: Conceptualization, Investigation, Writing – review & editing. RW: Conceptualization, Investigation, Writing – review & editing. MB: Investigation, Writing – review & editing. GG: Conceptualization, Data curation, Funding acquisition, Investigation, Methodology, Resources, Supervision, Writing – review & editing.
