# Supplementary material for: The patient’s sex determines the hemodynamic profile in patients with Cushing disease
Source: Front Endocrinol (Lausanne). 2023 Oct 11;14:1270455. doi: 10.3389/fendo.2023.1270455 (PMC10598757; doi:10.3389/fendo.2023.1270455)
Supplement: Supplementary file 1 [file Table_1.docx]

Supplementary Material

**The patient’s sex determines the hemodynamic profile in patients with Cushing disease**

Agnieszka Jurek*, Paweł Krzesiński, Beata Uziębło-Życzkowska, Przemysław Witek, Grzegorz Zieliński, Anna Kazimierczak, Robert Wierzbowski_,_ Małgorzata Banak, Grzegorz Gielerak

*** Correspondence:** Agnieszka Jurek, agnieszkajurek1@gmail.com

| Variables | Males (n = 12) mean ± SD / n (%) | Females (n=42) mean ± SD / n (%) | p-value |
| --- | --- | --- | --- |
| age (years) | 42.2 ± 13.3 | 40.8 ± 13.7 | 0.662 |
| BMI (kg/m^2^) | 30.5 ± 5.0 | 29.5 ± 6.9 | 0.399 |
| **comorbidities** |  |  |  |
| hypertension | 8 (66.7) | 27 (64.3) | 0.879 |
| dyslipidemia | 1 (8.3) | 8 (11.9) | 0.728 |
| diabetes mellitus | 3 (25.0) | 17 (40.5) | 0.328 |
| current smoking | 2 (16.7) | 4 (9.5) | 0.487 |
| **symptoms** |  |  |  |
| dyspnea on exertion | 3 (25.0) | 15 (35.7) | 0.487 |
| dyspnea at rest | 2 (16.7) | 6 (14.3) | 0.838 |
| dizziness | 6 (50.0) | 13 (31.0) | 0.223 |
| syncope | 0 (0.0) | 4 (9.5) | 0.267 |
| heart palpitations | 3 (25.0) | 20 (47.6) | 0.162 |
| peripheral edema | 5 (41.7) | 24 (57.1) | 0.343 |
| Abbreviations: BMI ‑ body mass index; SD ‑ standard deviation | | | |

**Supplementary Table S1.** Comparison between males and females – basic characteristics.
